# Sensors and Communication Simulation for Unmanned Traffic Management

**DOI:** 10.3390/s21030927

**Published:** 2021-01-30

**Authors:** David Carramiñana, Iván Campaña, Luca Bergesio, Ana M. Bernardos, Juan A. Besada

**Affiliations:** Information Processing and Telecommunications Center, Universidad Politécnica de Madrid, 28040 Madrid, Spain; d.carraminana@upm.es (D.C.); ivan.campana@upm.es (I.C.); luca.bergesio@grpss.ssr.upm.es (L.B.); anamaria.bernardos@upm.es (A.M.B.)

**Keywords:** UAV, drone, UTM, U-Space, C2, surveillance networks, agent-based simulation

## Abstract

Unmanned traffic management (UTM) systems will become a key enabler to the future drone market ecosystem, enabling the safe concurrent operation of both manned and unmanned aircrafts. Currently, these systems are usually tested by performing real scenarios that are costly, limited, hardly scalable, and poorly repeatable. As a solution, in this paper we propose an agent-based simulation platform, implemented through a micro service architecture, which may simulate UTM information sources, such as flight plans, telemetry messages, or tracks from a surveillance network. The final objective of this simulator is to use these information streams to perform a system-level evaluation of UTM systems both in the pre-flight and in-flight stages. The proposed platform, with a focus on simulation of communications and sensors, allows to model UTM actors’ behaviors and their interactions. In addition, it also considers the manual definition of events to simulate unexpected behaviors/events (contingencies), such as communications failures or pilots’ actions. In order to validate our architecture, we implemented a simulator that considers the following actors: drones, pilots, ground control stations, surveillance networks, and communications networks. This platform enables the simulation of the drone trajectory and control, the C2 (command and control) link, drone detection by surveillance sensors, and the communication of all agents by means of a mobile communications network. Our results show that it is possible to truthfully recreate complex scenarios using this simulator, mitigating the disadvantages of real testbeds.

## 1. Introduction

Unmanned aerial vehicle (UAV) usage has experienced a remarkable expansion in the last decades, thanks to key advancements in the technological domain. In particular, improvements in flight control and communications systems, an increase on flight autonomy—together with the reduction of UAVs’ dimensions—and decreasing prices have been vital to shift the drone ecosystem away from its original military-focused use cases. Currently, the drone market is undergoing a democratization process where enterprises and individuals can easily access drones and benefit from the competitive advantage they may provide.

Thus, drone usage is expanding to new use cases, such as precision agriculture [1,2], infrastructure inspection [3], parcel shipment [4], or even considered for urban personal mobility in a further future. In fact, according to the European Drones Outlook Study [5], the European commercial drone fleet is expected to grow rapidly, being formed of 400,000 hulls along with about 7 million private aircraft by 2050. Consequently, unmanned traffic is expected to become prevalent in the low-level and very low-level airspaces. 

However, the increasing drone usage has been followed by a growth in incidents caused by these aircrafts. For example, flights in the vicinity of airports cause major disruptions in its normal operations while increasing the risk of accidents for manned aircrafts [6]. Unauthorized flights have also occurred in densely populated urban areas with the consequent risk to the population on the ground. Other serious incidents include flying in sensitive and restricted areas such as nuclear power plants or military bases, intentionally or unintentionally. Finally, we cannot rule out in-air incidents caused by either malfunctioning aircraft or pilot’s errors that may lead to damage to people and property.

The operational concept defined for the safe use of drones seeks the total integration of manned and unmanned aircraft over the same airspace [7]. Nonetheless, traditional air traffic management (ATM) systems are highly dependent on the human factor, which conflicts with the inherent automation of drone operations. Apart from this, ATM systems would be unable to scale to cover the high number of operations that are expected in the future. Therefore, it is evident that it is necessary to develop new control systems that allow the efficient concurrent operation of UAVs while guaranteeing the safety of the rest of the aircraft and the citizens. These new systems are known as unmanned traffic management (UTM) systems.

Formally, a UTM system is a set of digitized and highly automated services designed to allow a high number of drones to access airspace safely and efficiently [8]. The definition of services to be provided to users within UTM is currently under discussion, with different potential architectures [9]. In all of them there will be a collection of services provided to the airspace users (drone operators and pilots) and to authorities (law enforcement, emergency, etc.) by either a central entity (i.e., an air navigation service provider, ANSP), or by a collection of UTM service providers (USPs), working collaboratively. A summary of the intended services to be provided is detailed in Table 1, taken (and summarized) from the European SESAR2020 U-Space documentation [10]. The table also shows the associated target time for services launch, in four phases called U1, U2, U3, and U4.

In other words, a UTM system is a system of systems where the aforementioned entities interact and exchange information to enable routine drone operations. These interactions can be divided into two large groups: pre-flight interactions and in-flight interactions. In the first group, the authorization process is vital so that the UTM system can be aware of the operations of drones and can guarantee its safety in advance. This process is initiated at the request of the operator by sending the description of an operation or flight plan to the USP. The USP verifies that regulation and airspace requirements are met and that there are no conflicts with other operations. If all conditions are met, the operation is authorized and registered in the UTM system to avoid later simultaneous operations in that area. In addition, all operations are also in-flight monitored. To do so, drones periodically send position information (also known as telemetry) to the USP. Additionally, this information may be merged with data from traditional ATM systems (i.e., radar and other air surveillance sensors tracks) and sensors from different drone surveillance networks. All these data allow to track drones and conflicting aircraft in real time, while monitoring the safety and conformance with the issued authorization and safety rules of all operations, generating alerts in case of deviations or risks.

Additionally, non-cooperative drone surveillance sensors may detect drones performing operations that have not been authorized and declared with the UTM ecosystem (non-cooperative drones). This information is key in some use cases, such as detecting unauthorized flights over critical infrastructures or real-time tactical conflict detection with cooperative drones or with manned aircraft. The prototypical example of this type of sensors is radar systems. Although traditional radar systems are not suitable for small aircraft, there are specific adaptations for RPAS (remotely piloted aircraft system) [11]. Moreover, visual techniques can also be used by means of cameras [12], acoustic techniques by means of microphone arrays [13], or radio frequency techniques detecting the emissions generated by the drone’s command and control (C2) link [14].

Other surveillance networks are composed by cooperative sensors that detect the position of drones from status messages sent by the aircraft themselves through a broadcast network. Contrary to the aforementioned sensors, the detection of an aircraft by means of cooperative sensors requires the presence of compatible equipment on the aircraft (cooperative drones) that, being active, periodically and automatically transmits its position. Within this group we find the ADS-B system (automatic dependent surveillance–broadcast) [15], widely used in commercial aviation. Moreover, a similar system designed for small aircraft and with a shorter range called FLARM system [16] may be used. Finally there is also the possibility to receive drones positioning through the Remote ID standard [17], part of the UTM functionalities, that allows the drone’s position broadcasting via WiFi, Bluetooth, and cellular communications. Although it does not require the presence of sensors, periodic sending of telemetry to the UTM system over public communication networks could also be considered as a cooperative surveillance system.

From all the above, it can be concluded that the operation of UTM systems depends on having ubiquitous, reliable, and low latency connectivity between all actors. The availability and integrity of communications and drone operations are quite often tightly interrelated. For instance, as a drone moves away from a communications node, connectivity might be interrupted prompting the drone to halt its predefined flight landing or returning to a known location.

We can distinguish three communication flows. First, there are air-ground communications (also known as C2 communications) that allow pilots to control and monitor drone operations from a ground control station (GCS). The second communication flow refers to the data exchange (telemetry and alerts) with UTM systems and it can be performed directly from the drone or from the GCS. These communications can be established using a wide variety of networks [18] from satellite networks (on large drones flying at high altitude), public mobile communication networks (for which special extensions are being developed [19]), ad-hoc Wi-Fi networks, long range IoT (internet of things) technologies such as ZigBee, to custom radio technology and specific communication protocols. Finally, vehicle-to-vehicle (V2V) connectivity is expected to become a key enabler of advanced detect and avoid (DAA) technologies [20] that would enable high density flight in urban areas.

In order to develop UTM systems with the aim to ensure the highest safety and security requirements, a comprehensive set of tests and evaluations is needed. These tests cannot only focus on drone mechanics to predict its trajectory but need to study several aspects: pilot actions and control through GCSs, drone navigation and control systems, communications between the different actors in the operation, and sensors networks. Moreover, it is not enough to analyze each aspect independently because, as mentioned before for communications, all of them are highly interdependent and emergent behaviors may occur with slight changes in one of them.

The most common way to evaluate the overall behavior of UTM systems is to perform live experimentation in scenarios that allow to generate the necessary information to excite the system. However, it is not possible to perform real tests at the scale that UTM systems are expected to operate, with hundreds or thousands of aircraft flying simultaneously. This would entail a very high economic cost, in addition to its difficulty as these tests are subject to meteorological variability, to the availability of equipment and personnel, and to the human factor. Furthermore, actual tests involving validation of safety in critical and emergency situations can lead to serious risk for equipment and people. For example, it will be necessary to evaluate the collision detection and avoidance algorithms between drones and manned aircraft, and this could require putting the aircraft at serious collision risk, which would not be acceptable.

As an alternative, we suggest the use of simulation techniques to perform reproducible, scalable, and exhaustive testing while reducing costs and evaluation time. We propose an agent-based simulation architecture (simulator, from now on) that allows reproducing the set of input signals required to excite an UTM system, i.e., authorization requests, telemetry messages, incoming tracks (continuous trace that by means of a unique identifier represents the consecutive positions of an aircraft) from sensors or surveillance systems, etc. Agent-based simulation is a subtype of discrete event simulation in which a series of entities, called agents, interact autonomously based on a series of rules. This simulation technique is useful to model complex systems that can be explained as an aggregate of these actors and is especially useful for the detection and assessment of emergent behavior.

The simulator is able to reproduce user-defined complex scenarios encompassing the actions of the main actors: pilots, drones, GCSs, surveillance and communications network; and the complete transmission chain followed by the information to the UTM system. This simulation is also linked to other external elements (e.g., weather, terrain morphology) that might affect the behavior of the previous actors constituting a common simulation environment for the architecture itself and for the UTM system. Finally, the proposed simulator is capable of simulating actions that can cause a change in the default or expected behavior of an actor including system failures, environment variations, or pilot’s decisions. These actions are known as contingencies and can occur both naturally during the simulation and by simulator user definition (as checking the reaction of UTM system to contingencies occurrence would be critical to assess its safety).

**Figure 1:**
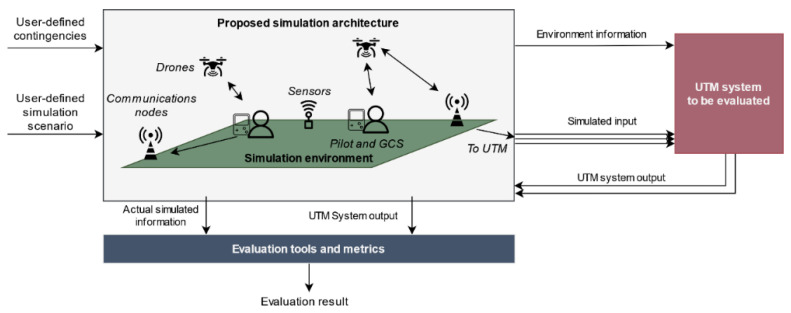
Proposed simulation framework and high-level unmanned traffic management (UTM) simulator integration.

By using these simulated inputs, it is possible to evaluate both pre-flight and in-flight stages by analyzing the output information provided by the UTM system and the actual actors’ simulated state. The proposed simulator is evaluation-agnostic in the sense that it does not define which metrics or tests must be performed to assess UTM performance. Alternatively, it provides a set of tools that enable their implementation by accessing information from the simulator and the UTM system. The information flow between the proposed simulator, the evaluated UTM system, and the evaluation tools is depicted in Figure 1.

## 2. Related Work

In the literature, there are numerous simulation proposals oriented to drone flight simulation modeling aspects such as mission planning and drone navigation and control. In [21], we find an analysis of the existing commercial tools such as X-plane, Flight Gear, JMavSim, or Microsoft AirSim. These tools allow realistically simulating drone flight and control in a synthetic three-dimensional environment that can be visualized by the user. This simulation level can also be accomplished using the simulator developed by the manufacturer DJI for its drones [22]. DJI simulator allows using the physical drone controller to pilot within the simulation environment. There are other tools devoted to modeling mission planning and GCS behavior like QgroundControl or MAVProxy. This last tool allows simulating C2 link and the interchanged messages between the GCS and the drone using UDP (user datagram protocol) and MAVLink protocols.

Simulation of C2 and V2V communications is presented in [23], where a simulation tool based on the Unity platform is proposed. This tool models aircraft specification, navigation, control, communications between different drones, and sense and avoid systems. Unlike previous simulators that were oriented to the simulation of a single drone, this one is designed to support simultaneous operations of several drones in order to analyze the operation of different sense and avoid algorithms. A multi-drone simulator is also proposed in [24], which presents a web architecture designed to simulate mission planning, execution, and monitoring of multiple UAVs within a virtual environment that can contain no-flight zones.

A multi-agent simulation architecture is proposed in [25] to test how traffic distribution algorithms affect drone operations. Aside from the usual flight planning and trajectory simulation capabilities, this architecture also allows simulating the coverage of a communications network used by the drones, by defining a scenario that geographically positions the communication nodes.

All the previous tools are self-contained and do not interconnect with UTM systems. If we focus on simulation oriented to UTM systems, the number of publicly available tools is very reduced. As a result of NASA’s developments to investigate UTM systems, a set of tools [26] has been created that allows defining drone trajectories as a list of waypoints and simulating in real time drone movements in order to generate position report messages (telemetry). This simulator also allows manual control in order to introduce contingencies in the trajectories. In addition, all the tools are integrated (e.g., telemetry messages are injected into the system) with the UTM system so that they can be used for testing and evaluating it through simulated scenarios.

To conclude, the available solutions do not offer a comprehensive simulation tool covering all the aspects that would be needed to evaluate a UTM platform effectively as will be detailed in the following section. In fact, no previous literature has been found for drone surveillance networks or sensors. Furthermore, simulators are not conceived to be integrated with a UTM system for evaluation (except the system proposed in [26]) and thus data exchange with UTM platforms is disregarded focusing only on C2 and V2V communications.

## 3. Comprehensive Simulation Scenarios for UTM Services Validation

Although the focus of this paper is the simulation of communications and sensors, it is not possible to do so (due to the interrelations explained in Section 1) without simulating comprehensive scenarios also covering drones, pilots, and the environment in which all these actors operate. Therefore, it is necessary to discuss which aspects related to both actors and the environment must be modeled to generate comprehensive simulation scenarios for UTM systems evaluation. It has to be noted that, from the systematic point of view, a detailed modeling of a given actor is only relevant insofar as the changes arising in that level of detail may affect the behavior and results of the UTM system.

We can start by identifying the necessary aspects related to drones, as they constitute the main actor within a UTM platform. A comprehensive simulator must be able to reproduce the trajectory followed by a drone as it executes a given operation (defined with a flight plan) but also taking into account other factors such as drone dynamics and meteorology. Drone trajectory is also affected by positioning errors suffered by the drone navigation system and control errors due to the drone’s autopilot, which should be taken into consideration too. In order to interact with a UTM system, a simulator must also be able to assign unique identifiers to each of the simulated drones (e-registration function). Finally, drones may also be affected by contingencies related to equipment failures, such as the loss of control (LOC), loss of GPS (LOG), and loss of battery (LOB).

However, drones operate at the command of pilots who are in charge of designing the drone operation, submitting a flight plan to the UTM system authorization process, and in some cases manually flying the drone. Our simulator is capable of generating both visual line of sight (VLOS) and beyond visual line of sight (BVLOS) traffic (flights) for different drones within a user-defined geographical area and time frame. Traffic generation also considers different flight priorities that might have an impact on authorization. Afterwards, the simulator emulates the submission of each flight plan to the authorization process. Some pilot decisions can be modeled at this point as contingencies. First, a pilot might decide to fly without submitting a flight plan to the UTM system. Then, it is also possible that the pilot decides not to respect the result of the authorization process performing the operation (flying) even when it has been rejected.

Within the flight phase, we find new pilot-related contingencies: the pilot may decide not to respect the approved flight plan and fly following another route or at a different time. Likewise, he can initiate actions such as landing or starting a return-to-home maneuver at any time. In addition, the pilot also decides if the drone will send information periodically to the UTM system (cooperative drone) or not (non-cooperative drone). Moreover, control of the aircraft is not perfect, and the final trajectory followed by the drone might slightly deviate from an ideal one, with a control error which will depend on the pilot’s skills or autopilot quality.

The C2 communications channel between the GCS and the drone should be simulated both in case of VLOS operations (usually consisting of a direct wireless communication link) and in BVLOS operations (typically making use of mobile communications networks). Through this channel, command sending to the drone and the reception of information about its status must be individually simulated. Furthermore, it should be possible to generate and simulate a contingency consisting of the loss of link (LOL) between the drone and the GCS. Simulating these aspects is vital as a message loss or link interruption can trigger important side effects such as trajectory changes or the disruption of the telemetry sending process. Regarding the GCS, its communications with the UTM system are also considered including the aforementioned telemetry relay process but also flight start and end notices, other alerts, etc.

In the case of non-cooperating drones, they need to be detected and tracked making use of non-cooperating surveillance sensors. So, to assess the capability of the UTM system to reduce the associated risk, we need to simulate both those sensors and their communications with the UTM system. Therefore, sensors positioning and range, target detection errors (false alarms, misses, sensor precision), information processing, and track sending are implemented in the simulator. In addition, cooperative sensors must also be considered taking into account the presence/absence in a given drone of the equipment required for state message broadcasting for each surveillance technology.

Finally, the communication between the actors and the UTM system would not be possible without simulating public communication networks. Thus, the simulator has the necessary mechanisms to simulate both fixed and mobile communications between the different actors, taking into account different types of network (3G, 4G, 5G) nodes forming a given architecture and the compatibility of each actor. To do so, it simulates basic forwarding and routing between those nodes and typical disturbances such as packet delays, losses, or bursts. In the case of mobile communications, the range of the nodes and the assignment of actors to each node are also considered. In relation to these last two actors (sensors and communication nodes), our simulator also models their possible failure as a contingency.

Regarding the environment in which drones operate, UTM systems are designed to work with real geographic coordinates. Therefore, the geographic space on which the simulation is developed is a representation of the real physical space using the WGS84 coordinate system. Moreover, the simulator also has a digital terrain model to reproduce real topography. Meteorological information may also be simulated or obtained from real weather predictions. Weather models include parameters such as temperature, pressure, and wind speed and direction. The simulator also includes a simplified airspace model with non-flight areas defined as volumes in 4-dimensions: area, heights and time. This model is dynamic and allows the creation of new non-flight areas in simulation time.

A comparison of the simulated elements in each of the existing proposals reviewed in Section 2 and the elements considered in our proposal is shown in Table 2.

## 4. Agent-Based Simulation in a Distributed Architecture

### 4.1. Simulation Approach

To model all the behaviors identified in the previous section, we will implement them as a composition of the interactions and states of all the actors using an agent-based simulation approach. As previously stated, due to its inherent down-top approach, agent-based simulation is of special interest for the discovery of emergent behaviors, which in our case is vital to guarantee the safety of UTM systems. Hence, each of the individual actors (drones, GCSs, network node, sensor) will correspond to an agent that will be modeled individually. These agents will autonomously update its state and interact with other agents at simulation time.

To do so, we propose a mixed closed/open loop, real-time, model-agnostic, and scenario-centered microservices simulation architecture that provides the necessary abstractions and mechanisms to easily model agents and execute simulation scenarios in a simple and reusable way.

Our current simulation architecture closes the management loop for pre-flight (strategic) operations, enabling testing of automated flight plan authorization by UTM and making simulated flights following amended flights proposed by it. Regarding in-flight control, the simulator is open loop. By open loop, we mean that although agents will take into account the states and interactions of other simulated actors, they do not consider UTM-provided tactical information such as alerts on nearby traffic. This is a current limitation of our simulator, to be addressed in future iterations.

Regarding time management, we are facing an eminently dynamic simulation that requires that all agents evolve synchronously through simulated time. In this sense, some evaluations (e.g., tracking and monitoring evaluation) could benefit from an accelerated time simulation approach, and also by the implementation of Monte Carlo simulation (repeated execution of experiments for statistical assessment). In the current version of our simulator, we implemented real time simulation instead of an accelerated time simulation due to limitations in the evaluated UTM platform (need to override timed processes with a common time reference). This is another area of future improvement of our proposal.

The proposed simulator is model-agnostic in the sense that it allows defining and simultaneously using multiple user-defined models for a given agent type. In addition, since we must model dynamic systems with random behaviors, the agents’ implementations will include stochastic parameters guaranteeing that the different simulation realizations are reproducible. All simulations are performed by defining reusable scenarios. These scenarios constitute the basic simulation unit, and their definition comprises all the required information to reproduce a simulation exercise, including details related to each agent, the models used to simulate them, the simulation environment, user-defined contingencies, and timing information.

### 4.2. Simulation Implementation: A Microservice-Based Architecture

The simulator is implemented within a microservice-based architecture that allows encapsulating the behavior of each type of agent in different microservices, so that the behavior of one agent can be modified without affecting the rest. In addition, the architecture is easily extendable as new agents could be added later simply by adding new microservices to the system. Microservices can be grouped in five different group based on their functionality: modeling services, pre-simulation services, simulation services, support services, and evaluation services. Thus, the operation of the simulation platform at high level is as follows:

In scenario definition time, by the simulator user through the simulator HMI (human machine interface):

1. Define a series of models for each actor.
2. Generate a scenario that determines all the actors involved in it and their configuration or model.

In simulation time, by the simulator platform itself:

3. Generate instances that simulate those agents from the selected model.
4. Agents’ instances would interact autonomously among them sending the needed information to the UTM system and receiving information from it.

Additionally, an interface with evaluated UTM service microservice is available which will enable networked communications between the evaluated UTM platform and the simulator. Both platforms are expected to be deployed within the same network so that delays are minimized. This service acts as a proxy between both platforms allowing messages exchange by translating data formats and communication protocols. UTM-bound messages will include authorization requests, track information from sensors, or telemetry information from drones whereas simulation-bound messages consist of UTM outgoing tracks with alerts and contextual information.

**Figure 2:**
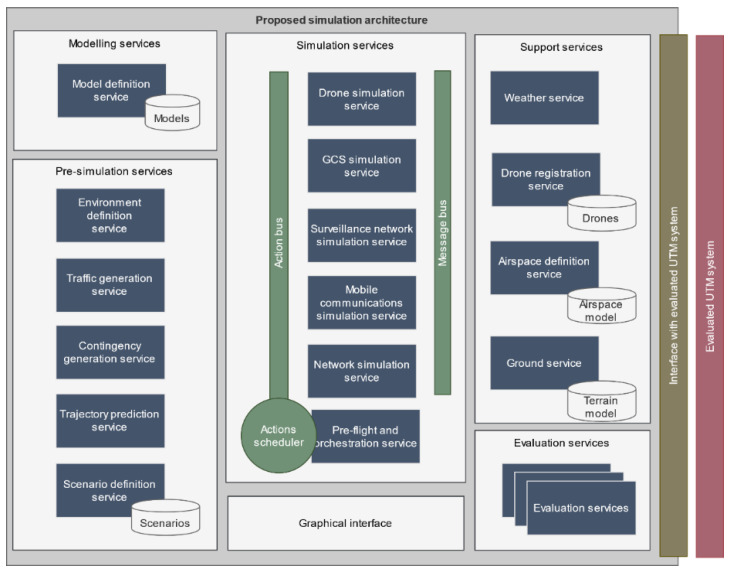
Proposed simulation architecture.

It is expected that each UTM system will require developing a compatible interface service. However, this interface decouples the simulator platform and the UTM platforms and therefore, there is no need to modify them synchronously. This microservice also collects UTM output information so that it can be analyzed within the multiple evaluation services, which in turn can also access the actual information from the simulation services. The complete simulator architecture is depicted in Figure 2.

This architecture was implemented using state of the art technologies for microservices architectures. Node.js was used as the runtime environment for all microservices, which were coded using Typescript. On top, Express web framework was used to implement REST (representational state transfer) communication between microservices, whereas Redis allowed implementing both communications buses. Persistent information is stored using MongoDB, a document-oriented database. Furthermore, microservices deployment were orchestrated using Docker virtualization containers.

### 4.3. Pre-Simulation Workflow: Models and Scenario Definition

Next, the functionality of those services in charge of defining the different simulation scenarios is presented. First, the model definition service, together with its associated database, allows defining and storing different simulation models for each simulation element: pilot behavior, drone types, sensor types, communication nodes, sensors, airspace structures, and weather. These reusable models are composed of two elements:

- A set of statistical properties and parameters that determine an agent behavior, which is persisted in the aforementioned database (named “model”).
- An associated programming class (named “behaviour_class”) in the corresponding simulation service that implements the modeled behavior and uses the previously mentioned parameters to reproduce agent behavior at simulation time.

This way, for a given type of agent, different behaviors can be defined as different behaviour_class implementations. Moreover, different sets of parameters (different models) can also be defined to alter the same behavior by linking them to the same behavior class. Models can be retrieved by other services to define the scenarios in generation time and to generate the appropriate instances of each agent.

Model information is used within pre-simulation services to allow the definition of a simulation scenario that is orchestrated by the scenario definition service, that also maintains a database to persist all scenarios. A scenario can be manually defined by the user or generated randomly by the platform using some user-provided information (simulation area and duration, number of flights, sensors and communications nodes density) and statistical models included in each of the services. Each scenario is composed of:

- A simulation environment which encompasses the definition of a mobile communications network as a series of geopositioned nodes, a surveillance network as a series of geopositioned sensors, an airspace definition as a set of no-flight zones or geofences, a model for a backbone fixed communications network, and a weather model. All these elements are defined using the environment definition service.
- A set of flights to be simulated. Each of them is specified through an expected flight plan, a drone, a GCS and pilot model. These flights are defined using the traffic generation service. As it is also necessary to predict drone trajectories from flight plans, this will be done by the trajectory prediction service.

**Figure 3:**
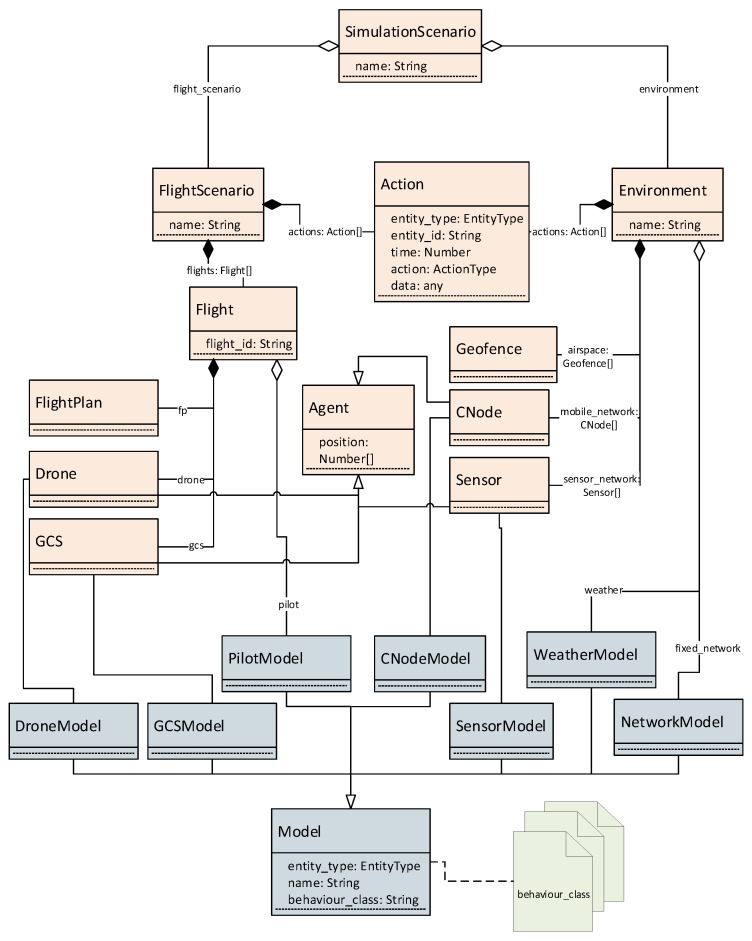
Proposed data model for simulation scenario definition (in orange), models definition (in gray), and associated behaviour_class files in each simulation service. For clarity purposes, the majority of classes’ parameters have been omitted.

Both elements include a list of actions (action class) that allows scheduling relevant user-defined events that are independent from the autonomous behavior of the actors, and that must occur during the simulation: flights start, flights end, and forced contingencies. By “forced” contingencies, we mean those that do not arise naturally from the interaction of the different actors in simulation time, but that are defined and programmed by the user in the scenario to guarantee their occurrence in order to be able to evaluate the UTM system reaction in their presence. The occurrence of these type of contingencies is modeled within the contingency generation service that allows their creation as actions that are appended to the action list. A detailed description of a scenario data model and its relationship with models defined in the model definition service is depicted in Figure 3. There, it can be seen that all agents are geopositioned and have an associated model, from those defined in the model definition service, which defines their respective behavior.

### 4.4. Simulation Execution

Simulation services constitute a distributed agent-based simulation environment. In this environment, the simulated agents update their state and interact in real time in an autonomous way. The scenario definition service database is the link between pre-simulation and simulation processes. The pre-flight and orchestration service allows configuring the simulation services (instantiating the different agents in each service using the class defined within the model) and support services according to the selected scenario stored in the database. The objective of this configuration stage is that once the simulation has started, the information flows autonomously between them. This service is also in charge of managing the flight authorization process and providing the actual state of the different agents to the evaluation services.

Two different communication buses have been envisioned to allow data exchange between agents in different microservices: a message bus and an actions bus. Through these two buses, the different events that determine the behavior of the agents are transmitted using a pub-sub messaging pattern. Following a discrete event simulation methodology, each event can result in changes in the internal state of an agent. Two main types of events are considered: messages or states exchanged autonomously between the actors (message bus), and actions programmed manually by the user that can significantly modify their behavior (actions bus).

The message bus can be divided into different logical channels through which the messages, exchanged autonomously between the different actors, circulate in the form of events that can modify the behavior of other actors. In each separate logical channel, this bus exposes to the end user a pub-sub messaging interface with hierarchical topics that allows implementing critical interactions such as mobile and fixed communication channels or the ground-to-air communication channel. Furthermore, thanks to the internal logical separation, it is also possible to use it to distribute information of changes in states (e.g., change of position) of the agents between the different microservices. As for the action bus, it allows sending the different orders (start of flight, end, etc.) and pre-programmed contingencies to the agents. Each action is issued as a properly timed event in an action planner that is controlled by the orchestration service. These actions can be directed to all services (thus configuring the start, pause, or resume of the simulation), groups of agents, or specific agents.

As it can be seen in Figure 2, a simulation service was built for each agent, except for pilots, as their impact in our architecture is limited to the introduction of contingencies that are generated in the pre-simulation phase and are treated in the form of actions. The communications network has been separated into two microservices following a two-level network model:

(1) an access level simulated by the mobile communications simulation service, and
(2) an aggregation/backbone level, which manages all traffic across mobile nodes and to the UTM system, which is simulated by the network simulation service.

More complex network models might be simulated by including more aggregation agents, our simulator, in principle, being agnostic with respect to the underlying network topology.

Each simulation service is implemented using a common architecture that allows the execution of the classes implementing the behavior of each agent as defined in each model (behaviour_class). They are also connected to both communication buses and receive and send events to the appropriated agents. They also provide timing utilities so that the processes defined for each model can be run periodically or at a given time.

The interactions among the services to simulate a scenario work as follows. First, the user selects a previously created scenario, and the pre-flight and orchestration service configures all agents and services according to the models identified in that scenario. Then, it submits the different flight plans to the authorization process and sets those approved (or affected by a contingency) to be simulated. After that, the simulation starts at user request and an event is generated with a start order that is sent to all the simulation services through the actions bus. From this moment on, all the agents are activated, and their internal processes of status update started. At the same time, the actions bus and message bus are also activated. When the agents start working, the exchange of messages in the form of events between the different agents through the message bus begins. At the same time, the periodic sending of information from the simulation agents towards the UTM system also starts. This information is also compiled into messages that are transmitted across the simulated network (mobile communications simulation service and network simulation service) using the message bus. Then, it is captured by the interface with evaluated UTM service microservice, translated to UTM data formats, and finally forwarded to the UTM platform. In parallel, the action planner sends the actions in the planned time instant to the actions bus that makes them arrive to the corresponding simulation service, so that the agents act accordingly modifying their behavior.

**Figure 4:**
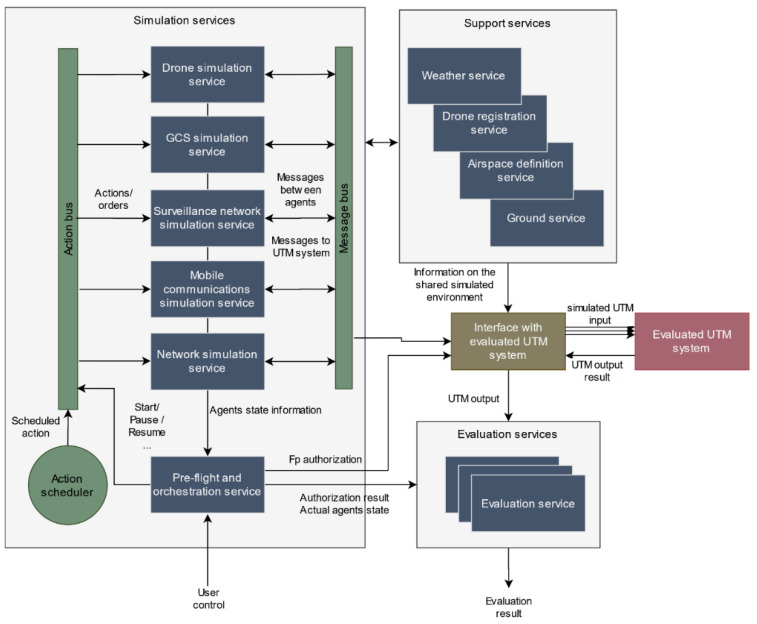
Simulation services interaction in simulation time.

As the UTM system receives the different input messages, it generates outputs that are available for the analysis of the evaluation services. At the same time, current agents’ state can be retrieved at any time through the pre-flight and orchestration service that has access to it through a REST interface with the different simulation services. With this information, the evaluation services can produce their evaluation metrics, achieving the ultimate goal of our platform. This simulation process is depicted in Figure 4.

Finally, the environment on top of which the simulation is developed is defined by the meteorological service, which is in charge of providing weather information (either real by accessing openweathermap or user-defined), the airspace definition service that provides information of non-flight zones, and the ground service that allows access to a digital terrain model (using TanDEM-X 90m data [27]). Additionally, the drone registration service manages the UTM identifiers allowing mapping them together with the drones in simulation to retrieve their information. To do so, it implements a functionality that mimics the registration functionality of a UTM system. These microservices model and share the simulation environment with the UTM platform, so it may adapt UTM operation to the rules, airspace, terrain and weather.

## 5. Proposed Simulation Models for Key Elements

With the simulation framework as starting point, we are going to discuss some of the most relevant models proposed to generate scenarios to simulate each agent.

### 5.1. Scenario Definition

**Figure 5:**
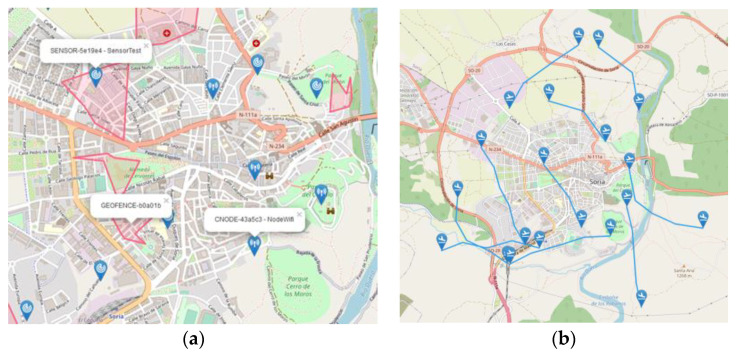
Example of automatically generated simulation environment (**a**) and flights (**b**).

The different elements composing a simulation scenario have been identified in Section 4.2. Therefore, we are going to focus on describing some specific elements. First, we have to remember that all agents must be georeferenced. Additionally, we must be able to represent geographical shapes to define no-flight areas, flight plans, etc. To do so, the design decision is to use the GeoJSON [28] standard that allows defining points, lines, and polygons using JSON objects. Figure 5a, shows a randomly generated simulation environment with a series of communication nodes, sensors, and geofences whose position/shape is defined using this standard.

Regarding the description of drone operations, we defined a flight plan as a succession of waypoints that allows to unequivocally define the drone’s trajectory. Each point will have an associated series of additional height and time constraints. In order to compute this information taking into account both user-defined restrictions and drone dynamics, an operation planning process must be performed, as explained in [29]. This also includes a terrain avoidance step which uses information from the ground service. In addition, a flight plan will also have an associated unique identifier and a state to model the different steps and results of the authorization process. The identifying information (e-registration) of the drone to be used for the operation should also be included along with the type of operation (VLOS/BVLOS) and the priority of the flight plan. An example of a collection of automatically generated operations is provided in Figure 5b.

Finally, the contingencies that were already identified in Section 3 (LOL, LOB, LOG, LOC, and sensor/cnode failure) can be added to a simulation scenario in the form of scheduled actions as defined in Section 4.2. Some additional pilot behaviors can also be added at this stage as action objects including:

- changing control mode from automatic to manual and vice versa,
- stopping and resuming an operation,
- starting a return to home operation, and
- deviating from approved flight plan by flying a different operation that can be included as a secondary flight plan.

### 5.2. Drone Control, Navigation, and Trajectory.

The drone’s trajectory is simulated from the flight plan defined in the pre-simulation phase. This trajectory is completely generated before starting the simulation and it is only in case of modifications on the trajectory (due to pilot actions or reactions to contingencies) that new requests to the trajectory prediction service are made at simulation time. We focused our efforts on the simulation of multirotor drones using the trajectory calculation engine presented in [30]. This engine uses as a basis a formal language for defining intentions for quadcopters (the so called QR-AIDL, quadrotor aircraft intent description language) presented in [31]. It consists of a series of differential equations that model drone dynamics and that are constrained using the flight plan decomposition with QR-AIDL language. To model the drone, it requires the following input information: minimum, maximum, and average speed, aircraft mass, maximum power, maximum thrust, and battery endurance. This information is included in all drone model instances (DroneModel data type). Additionally, it also considers weather effect on the trajectory. To do so, the weather service provides the required information either using a user-provided weather model or querying external services to retrieve real data in the flight area. The trajectory obtained from this service is sampled and consists of a set of points that completely define the dynamic state vector of the drone: timestamp (referenced to the beginning of the flight), 3D position and speed, altitude, and remaining battery.

Taking this predicted trajectory as a starting point, we then considered the effect of the control drift either by the pilot or by drone’s autopilot and the navigation error. Control error (particularly in the manual control case) is time correlated. We modeled it by generating a random path that will allow us to slightly deviate from the original trajectory. By applying a linear transformation similar to the one of a constant acceleration linear movement to a Gaussian random walk, we obtained the following model Equation (1) that allows updating simultaneously both position and speed:

(1) $$\left[\begin{array}{c} \Delta x\left[n\right]\\ \Delta v\left[n\right] \end{array}\right]=f\left[\begin{array}{cc} 1 & T\\ 0 & 1 \end{array}\right]\left[\begin{array}{c} \Delta x\left[n-1\right]\\ \Delta v\left[n-1\right] \end{array}\right]+\sqrt{1-f^{2}}\left[\begin{array}{c} \frac{T^{2}}{2}\\ T \end{array}\right]a_{x}$$

where Δ*x* and Δ*v* are position and speed drifts, *T* is the time between two consecutive trajectory points, *a_x_* is the noise acceleration that causes the deviation modeled as $a_{x}\sim N\left(0,\sigma_{ax}^{2}\right)$, and *f* is the correlation factor. These two last constants would depend on whether the control is automatic and manual and should be defined in their corresponding models (DroneModel or PilotModel) in the model definition service.

Drone positioning using GNSS (global navigation satellite system) systems is subject to multiple sources of error: multipath propagation, clock errors, atmospheric propagation disturbances, etc. The drone control system (either pilot or autopilot) uses the position obtained from GNSS systems in a closed-loop feedback system that allows it to guide maneuver execution. These control systems smooth high frequency navigation noise components but have no observability of low frequency errors (such as biases or slow drifts). If we neglect high frequency errors, the kinematic state we calculate after applying the drone control error model in (1) would not correspond to the actual state of the drone, but to an estimate of the state already including this low frequency navigation error. This estimate would become the basis for the drone telemetry calculation, as well as for the simulation of the rest of cooperative sensors.

On the other hand, non-cooperative sensors detect the drone in its real position which, in a simple approximation, would correspond with the estimated position subtracting the navigation error. We model the navigation error in each of the three dimensions, as a time-correlated Gaussian additive error with zero average.

**Figure 6:**
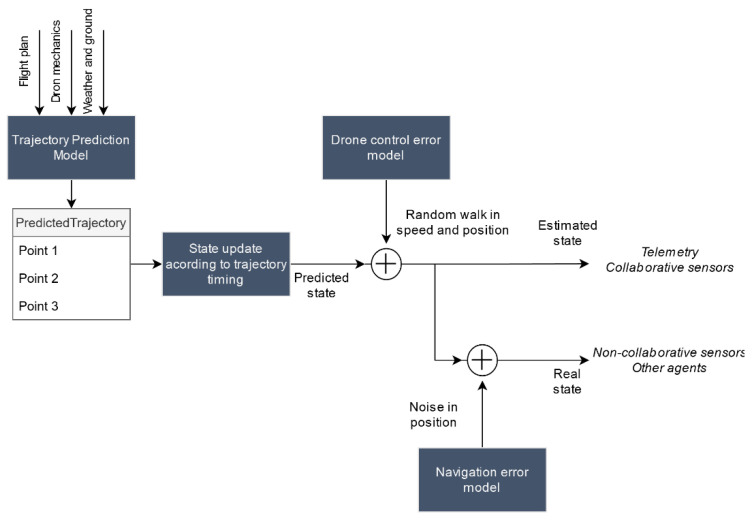
Drone position simulation process.

This process is depicted in Figure 6. Although it is a simplification of the actual navigation and control loops, this process is realistic enough to enable the assessment of tracking and conformance monitoring functions within UTM. After the position is updated, the real and estimated states are broadcasted through logical channels of the message bus so that this information can be used by other agents.

In the previous paragraphs we described the drone’s behavior in the absence of contingencies. However, the planned trajectory can be modified after receiving commands through C2 command (from the GCS) or by receiving contingencies through the actions bus. In either case, the predicted trajectory would be recomputed according to the following expected typical drone reactions:

- LOL (loss of link) or RTH (return to home) order from GCS. A straight-line trajectory to the take-off point would be performed.
- LOG (loss of GPS) or LOB (loss of battery). An immediate landing would be performed.
- LOC (loss of control). A random trajectory using a random walk would be simulated until battery exhaustion.
- Stop/resume order from GCS. Trajectory would stop and drone would hover in place until it is resumed.
- Change to manual/automatic order from GCS. It would change drone control error model parameters to the appropriate ones.
- Deviate flight plan from GCS. A new trajectory would be computed and simulated from the drone’s position to the starting point of the new trajectory and would continue by simulating the new one.

### 5.3. Communications Simulation

**Figure 7:**
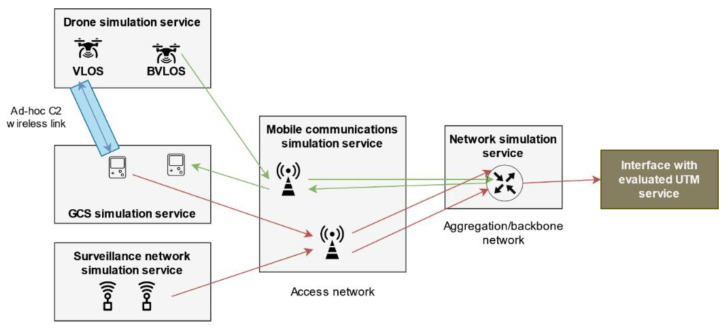
Simulated network architecture. Beyond visual line of sight (BVLOS) C2 traffic is shown in green, VLOS C2 link in blue, and UTM-bound traffic in red.

Public communication networks are modeled using a simple communication hierarchy integrating two different types of agents: mobile communication nodes, which will be simulated as multiple agents (each base station is an independent agent) in the mobile communications simulation service, and a fixed network that interconnects all the agents among them and with the UTM system. The fixed network is modeled through a single aggregation agent, within the network simulation service. Following these considerations, we can define our simulated network architecture in Figure 7, which is completely hierarchical. All simulated information traffic generated by drones, GCSs, or sensors accesses the simulated network through the different mobile nodes and their messages are then sent to the aggregation network. More complex network models might be simulated by including more aggregation agents, our simulator, in principle, being agnostic with respect to the underlying network topology. It is important to differentiate between the message bus and action bus messages, used to run the distributed simulation and synchronize agents’ behavior, and these simulated information messages, which are simulated counterparts of actual communications present in a real UTM system.

Once in the aggregation network, simulated information messages can be forwarded to the interface with evaluated UTM system (traffic flow shown in red in Figure 7) in case they are addressed to the UTM system (sending telemetry, tracks, alerts, etc.) or back to the mobile communications network (in green) if the traffic has a drone or a GCS as destination (ground-to-air channel in BVLOS flights). In other words, the simulated network can carry simulated traffic in both directions. On the other hand, the C2 communication channel for VLOS operations (in blue) is established through an ad-hoc, independent, wireless channel between the drone and the GCS, to be explained in Section 5.4.

In order to forward traffic across the different microservices according to the previously defined architecture, we implemented a three-level communications stack simulating the usual TCP/IP (transmission control protocol/internet protocol) tower levels: physical level, link level, and network level. First, the network level ensures that packets follow the correct end-to-end route between different services. To do this, it models packets as objects with the following parameters: protocol (robust/not robust), timestamp, source and destination services, source and destination agent within each microservice, and a payload with the information to transmit. Then, the link level takes care of packet routing between agents within the same network segment: actor-mobile network, mobile network-fixed network, fixed network-UTM interface. At this level, we model the packet in a similar way to the previous one: a payload and two 2-tuples (service-agent) that define the source and destination of the packet within the current network segment. Due to the low complexity of the network architecture, manual routing tables have been implemented to calculate the next hop (link level addresses) from the network level source and destination addresses. Finally, we find the physical level that is in charge of transmitting the packets and that has been implemented making use of a separated logical channel of the message bus. By using this separate logical channel, it is possible to emulate traffic forwarding by publishing messages (sending packets) and subscribing agents to the appropriate topics (receiving packets).

**Figure 8:**
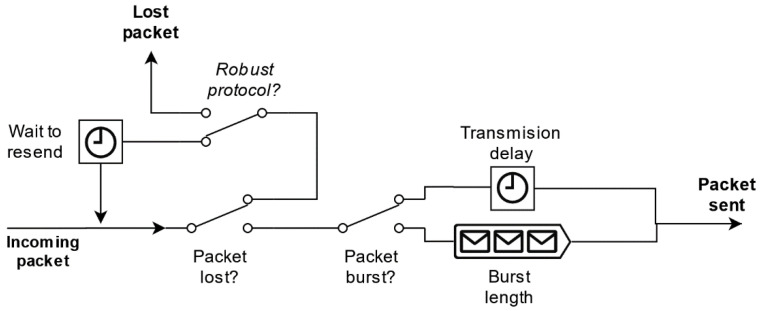
Perturbance simulation processing queue in each communications node.

Before sending a package through the message bus, each node simulates network disturbances: packet loss, packet burst formation, and packet transmission delay. According to our tests in the field, UTM-bound traffic from drones, sensors, or GCSs is practically negligible compared to other users’ traffic, as UTM-related messages are generally short (<1Kb) and do not have too high frequency (i.e., around 1 Hz). Therefore, we have modeled network parameters as dependent of a stationary network load status, determined not by the simulated UTM-related traffic but by the rest of the communication network users. To implement these disturbances, each communication node consists of a communication queue, as shown in Figure 8, that groups all received traffic, simulates the disturbances, and routes it to the next node. Each disturbance has been modeled using stochastic models (packet loss rate, packet burst rate, mean burst length, mean transmission delay, etc.), and an independent realization of each model is drawn to generate the corresponding disturbance for each packet. These parameters are included in both CNodeModel and NetworkModel and can be defined by the user.

Once we are able to forward traffic, we can analyze the specific behavior of the mobile communication nodes. We must take into account that a packet must only be sent by one node. Therefore, it is necessary to simulate the process of assigning a mobile communications node to each agent connected to the mobile network. This assignment depends on two important factors that we will simulate: the radio link between agents and the presence of different types of communication technologies. Starting with the communication technologies, each model of a communication node has assigned a communication type (WiFi, 3G/4G/5G mobile communications, etc.). At the same time, the drones, sensors, and GCS agents’ models detail their compatible communication technologies. As for the radio range between agents, we are going to implement a simplified coverage model. For each communication node, we define its maximum horizontal isotropic range and vertical range. If multiple nodes fulfil these requirements, the node closest to the agent is chosen. This process is performed in an orchestrated way among all agents using drone/GCS/sensor state update messages to know their position.

### 5.4. GCS and C2 Links for VLOS and BVLOS Operations

As previously explained, two possible C2 channels have been considered:

(1) A channel established directly between the GCS and the drone that is used in VLOS operations.
(2) A channel that establishes communication using the public mobile communications network, to be used for BVLOS operations management.

Over this channel, the drone periodically sends to the GCS telemetry messages including the following information: battery level, 3D position and speed, altitude, and alerts related to drone status (LOG). This information is then forwarded to the UTM by the GCS. The other existing interaction between the GCS and the UTM system is related to start/end of flight messages, including the authorized flight plan identifier. In parallel, the GCS continuously sends control commands to the drone. These commands can indicate that the drone must continue with mission execution, or they may schedule major changes in the drone flight plan due to the reception of action messages through the action bus. These last messages correspond to scheduled events such as flight start/finish and pilot actions as those defined in Section 5.1.

The implementation of the BVLOS channel is an example of communication through the mobile network as explained in Section 5.3. In this way, both the drone and the GCS will be assigned to a base station that will serve traffic consisting of the aforementioned state and control messages encapsulated in a simulated communication packet. Apart from this, the VLOS case requires the establishment of a new logical channel in the message bus for each drone-GCS pair allowing the direct exchange of events between the two agents. As it is a wireless communication link, the availability of this communication channel demands that the GCS and drone be in their respective coverages. This coverage is defined within the GCS model by a maximum vertical range and a maximum horizontal range in meters and is checked, once again, using the periodic drone state messages and the own GCS position. If the drone is out of range, command messages stream is stopped, and “received” state messages from the drone are disregarded.

The loss of link (LOL) situation is detected by both agents as they detect that no new state or command messages arrive. This situation can also be generated manually (simulating communication equipment failure) when the GCS receives a LOL contingency message through the action bus.

### 5.5. Surveillance Network

Two main parameters have to be considered in order to model sensors within surveillance networks. First, we have to define whether a sensor is cooperative or non-cooperative. Additionally, for cooperative sensors their specific technology (i.e., ADS-B, FLARM, etc.) needs to be defined. The second parameter to be defined is the sensor coverage, again based on a simplified model based on maximum horizontal and vertical ranges.

**Figure 9:**
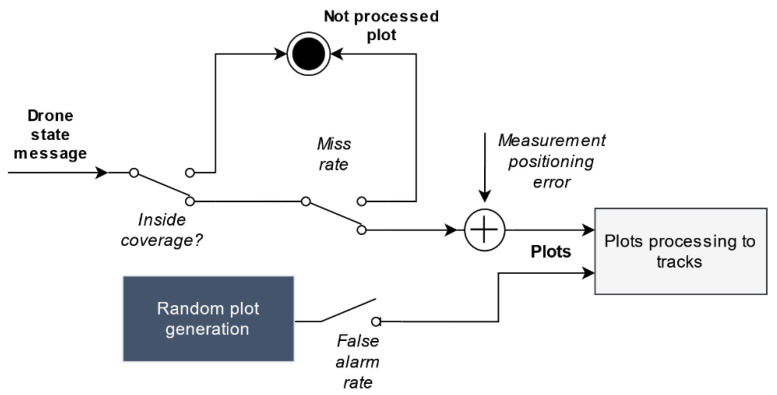
Non-cooperative sensor plot generation process.

Starting with non-cooperative sensors, each sensor receives the periodical drone state update messages (received through the message bus) that include their real position and checks if it is inside the sensor’s coverage. Three stochastic processes are used to generate plots from actual drone states, as depicted in Figure 9. First, the probability that a sensor does not detect the drone (miss rate) is considered. For actually detected drones, an additive measurement error is incorporated into real drone position in order to derive plot measures. In our current simulator, measurement errors are modeled as white Gaussian processes, but more complex models including biases, correlated noises, etc. could be easily incorporated. Finally, the sensor agent can also simulate false alarms within the sensor range, according to a sensor-specified false alarm rate. These stochastic processes parameters are specified in SensorModel objects, part of the simulation scenario specification.

In the case of cooperative sensors, drone detection depends on it transmitting its position voluntarily (cooperative drone). To simulate this transmission, a logical channel was implemented within the message bus. There, drones publish their estimated position in a series of hierarchical topics that corresponds to the types of collaborative equipment (transponders, emitters) they have installed. Collaborative sensors subscribe to the corresponding topics depending on their type, and they check if the received position is within the sensor’s range. If that is the case, a new plot is created.

Finally, sensors transform plots into tracks that are then sent to the UTM system. A track contains:

- a sensor identifier,
- the type of sensor that has generated the track,
- a unique track identifier,
- track reference time,
- estimated drone position,
- estimated drone speed, and
- drone identifier (if sensor is cooperative).

**Figure 10:**
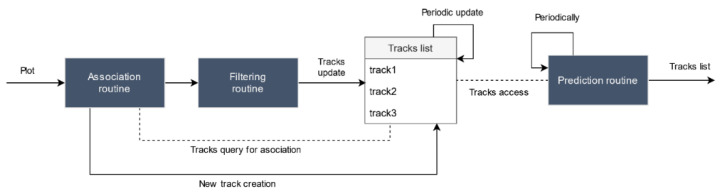
Sensor track processing schema.

To generate tracks, a classical monosensor tracking chain is simulated (although more complex data processors might be easily incorporated by modifying the sensor agent). Three processes are simulated within each sensor, as shown in Figure 10. First, each plot is associated to an existing track or a new track is created otherwise. Then, the incoming plot is filtered to reduce measurement errors through techniques such as Kalman filters, and the corresponding track is updated with the new information. Finally, all tracks positions are periodically predicted to a common time instant and sent to the UTM system.

## 6. Validation

Next, we focus on the validation of the simulation platform. The focus of our simulator is to enable the assessment of complete UTM platforms and more specifically, the discovery of potential negative emergent behaviors on UTM systems. Therefore, the validation of each of the individual agent models is not so relevant. It is much more important for us to check that the proposed platform allows simulating complex scenarios in which multiple agents interact, in presence of contingencies, while generating UTM input sources in a coherent way. Once the simulation architecture has been validated, refinement of individual agent simulation models is always possible, and in fact, the agent-based simulation approach nicely supports this iterative improvement of individual models. In any case, some of the most critical simulation models have been validated in previous publications with real flights, as those related to trajectory prediction and telemetry communication in [30,32].

**Figure 11:**
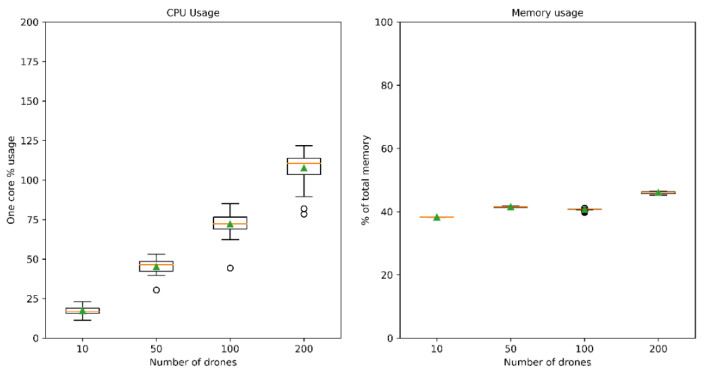
Box plots showing relative CPU and memory usage of the all simulator microservices during real-time simulation encompassing 10, 50, 100, and 200 drones. CPU usage limit is 200% as two cores are available. Average values are shown in green.

This system-level validation was performed using the simulator implementation described in Section 4.2. Simulation tests with hundreds of drones and dozens of sensors and communication nodes were performed, involving all types of contingency combinations. These tests were carried out using a modest hardware platform (2 core, 2.4 GHz, Intel Core2 Duo E6600 CPU from year 2006 and 6 GB of RAM) and show that the proposed platform scales well with the number of simulated entities without reaching computational bottlenecks, as can be seen in Figure 11. In addition, no relevant simulation-induced delays were detected as the number of entities increases. Moreover, entities usually send information to the UTM system each second which dampens the final impact of possible simulation delays.

This scalability is due to some design decisions such the distributed simulation among different microservices as well as drone trajectories precomputation (as explained in Section 5.2) which allows performing the CPU-intensive trajectory-prediction process only when the expected trajectory changes. Scenarios with a greater number of entities could be easily simulated either scaling vertically by using a more powerful hardware platform, or horizontally by distributing microservices between a set of locally-networked, time-synchronized computers. Multiple instances of a given microservice may also be deployed to increase performance.

Next, we show the correct operation of the platform in three example scenarios, involving just a few agents for clarity purposes. In order to generate the following graphs, an evaluation service was implemented to retrieve agents’ state and the information provided to the UTM system using platform-provided tools.

### 6.1. Drone Trajectory in Presence of Contingencies

**Figure 12:**
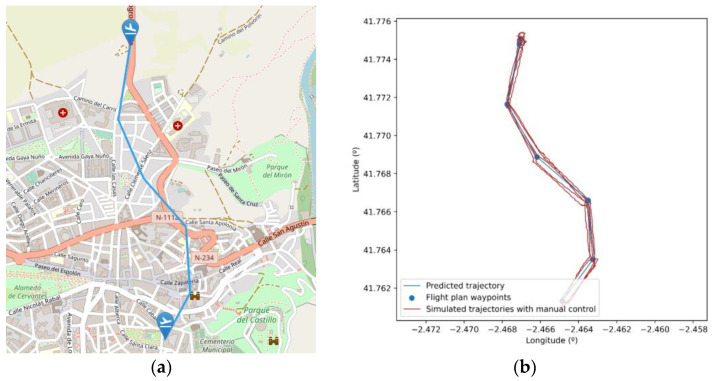
(**a**) Flight plan simulated in the proposed scenario. (**b**) Trajectory followed by the drone (in red) in five different realizations using manual control model.

The first scenario (depicted in Figure 12a) was built to show the results of the proposed drone control and navigation models, trajectory changes in presence of contingencies, and the generation of programmed contingencies. It consists of a single flight plan, defined to be flown manually by the pilot. Regarding the simulation of the control error caused by the pilot, we can see in Figure 12b that the simulator is able to generate different drone trajectories, deviating from the predicted path (in blue) by using the proposed model. The graph shows correlated deviations from the expected path (5 different realizations in red).

**Figure 13:**
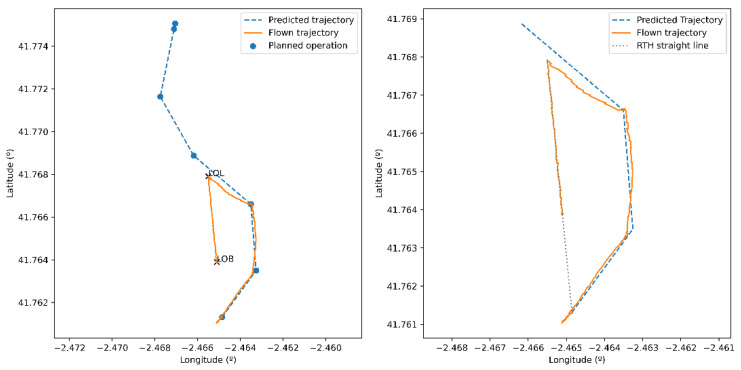
Comparison of the expected trajectory and the flown trajectory. The location where both contingencies happened is annotated on the left figure.

**Figure 14:**
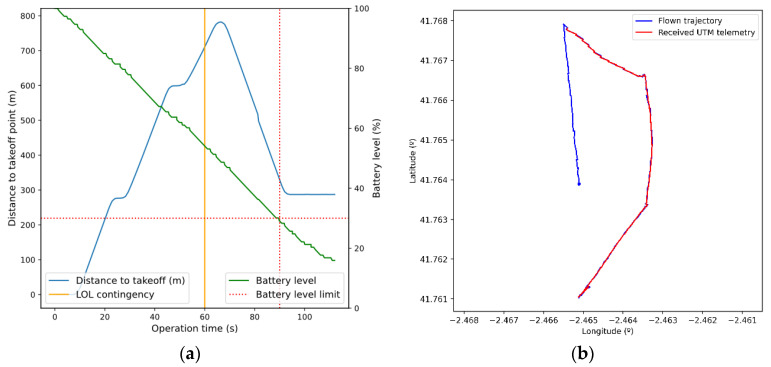
(**a**) Distance to the takeoff point and battery level time evolution. (**b**) Comparison of the simulated flown trajectory with the UTM-received positions.

Using the previous scenario as a base, a forced LOL contingency (loss of ground/air communications link) was scheduled after 60 s of flight. As a result, we can see in Figure 13 the simulated drone trajectory. Initially, the drone follows the associated flight plan slightly deviating from it due to the manual control model. However, after the second waypoint the expected operation suddenly halts, and a RTH maneuver is started. This is caused by the programmed LOL contingency. As expected, the GCS agent received from the action bus the forced contingency and has stopped the simulated C2 link. This was detected by the drone who automatically started a RTH maneuver with automatic control (now, the simulated trajectory deviates very little from the expected one which is a straight line to the takeoff position, as the autopilot is controlling the drone). However, in the course of the operation back home, the simulated drone battery level drops below a minimum threshold (30%, see Figure 14a) set within the drone model. A LOB contingency arises, which implies that the drone must land immediately. Thus, the automatic maneuver is stopped and a new one is initiated, which makes the drone land on the same position it is in that moment, without reaching the takeoff point.

In parallel to what has already been explained, the GCS sends telemetry information to the UTM system periodically until the C2 link stops. Figure 14 shows the trajectory actually flown by the drone, which differs slightly from the telemetry information received by the UTM system. This is due to the fact that the real position of the drone and estimated position in telemetry messages differ, due to navigation error, as explained in Section 5.2.

### 6.2. Drone Detection Using Cooperative and Non-Cooperative Sensors

**Figure 15:**
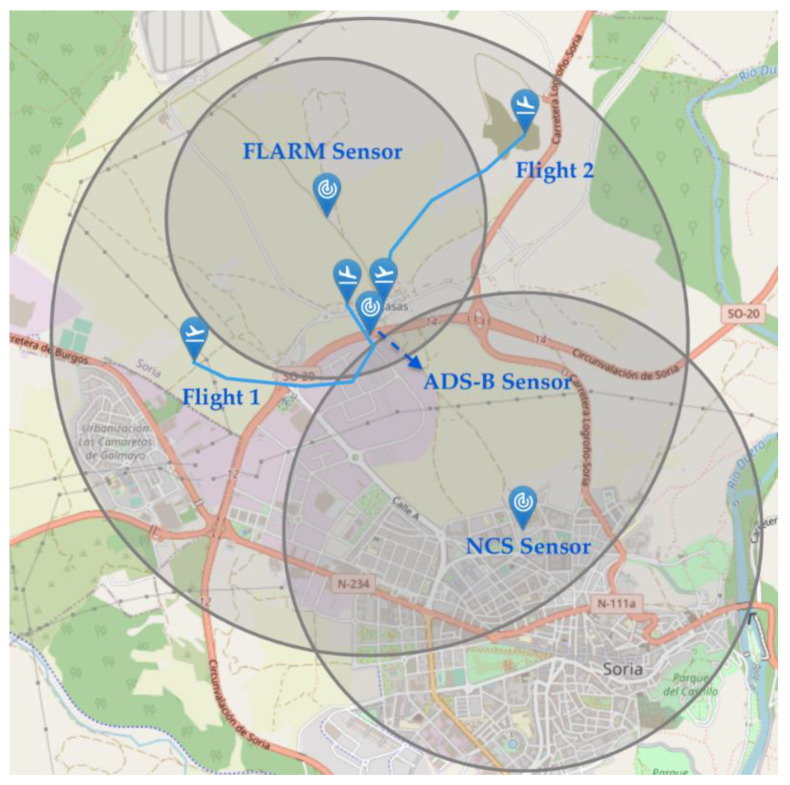
Scenario 6.2 with two different flights and three sensors.

This second scenario seeks to check the correct behavior of the cooperative and non-cooperative sensors network. For this purpose, a simulation environment was generated that consists of three different types of sensors: a FLARM cooperative sensor, an ADS-B, and a non-cooperative sensor. The positioning of these sensors and their detection range can be seen in Figure 15. In this environment, we simulate two flights whose flight plans can also be seen in the same figure. Flight 1 is operated by a cooperative drone which is equipped with a FLARM emitter. On the other hand, Flight 2 has equipment for the FLARM and ADS-B sensors. However, this second operation is marked as non-cooperative by simulating the pilot’s decision not to share his position.

**Figure 16:**
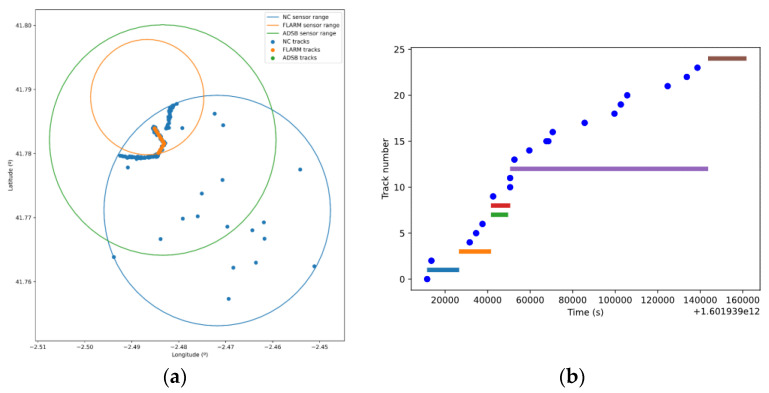
(**a**) Sensors range along with the position sent to the UTM system by each sensor. (**b**) Time evolution of the track numbers sent by the non-cooperative sensor.

This scenario was completely simulated without introducing contingencies. The tracks sent to the UTM platform are depicted in Figure 16a. We can first observe how the spatial detection limits of the sensors are respected. Thus, all the tracks sent by the FLARM sensor are limited to the interior of its coverage area. The non-cooperative sensor (NC sensor in the figure) has been able to detect, as expected, both cooperative and non-cooperative drones along their trajectory within the coverage area. Furthermore, we can also observe how this sensor simulated false detections as seen in the randomly distributed plots within the coverage area. Missed detections, resulting in monosensor track reinitialization, were also simulated. This can be seen in Figure 16b, where track numbers (identifiers) are depicted. Longer horizontal lines are related to real drones’ identifiers, which change from time to time due to the aforementioned track reinitialization. In this same figure, blue points are related to false alarm tracks, typically with very short life. Apart from this, cooperative sensors only detected cooperative drone with compatible equipment; it is the case of flight 1 and the FLARM sensor. For the same reason, flight 2 was not been detected by any of the cooperative sensors in spite of carrying FLARM and ADS-B equipment, since it is not cooperative.

**Figure 17:**
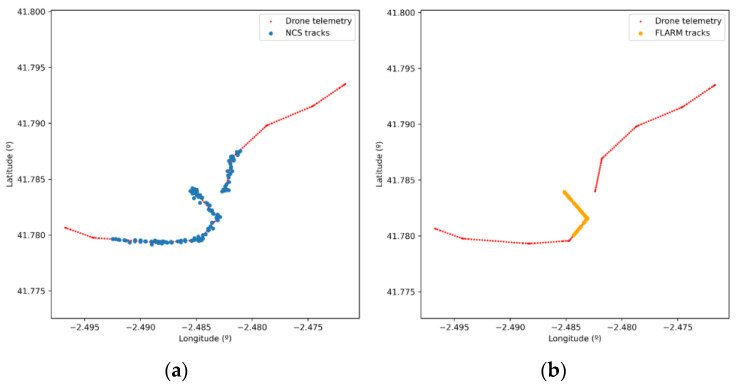
Comparison of the position received in the UTM system for both flights from non-cooperative (**a**), cooperative (**b**) sensors and telemetry from drone.

Finally, position information obtained from each type of sensor can be compared. Cooperative sensors obtain the position from drones’ navigation systems. Thus, we can see in Figure 17b how the information sent to the UTM system by the sensor is the basically the same as the telemetry information sent by the drone (codification errors due to channel quantization may arise). The non-cooperative sensor (NCS) measures the real position of the drone, and therefore there is a divergence with telemetry, due to both navigation error and NCS measurement/tracking error. Hence, the information sent by the non-cooperative sensors to the UTM system is different from the information received from the drones through telemetry, as we can see in the Figure 17a.

### 6.3. Public Communications Networks

**Figure 18:**
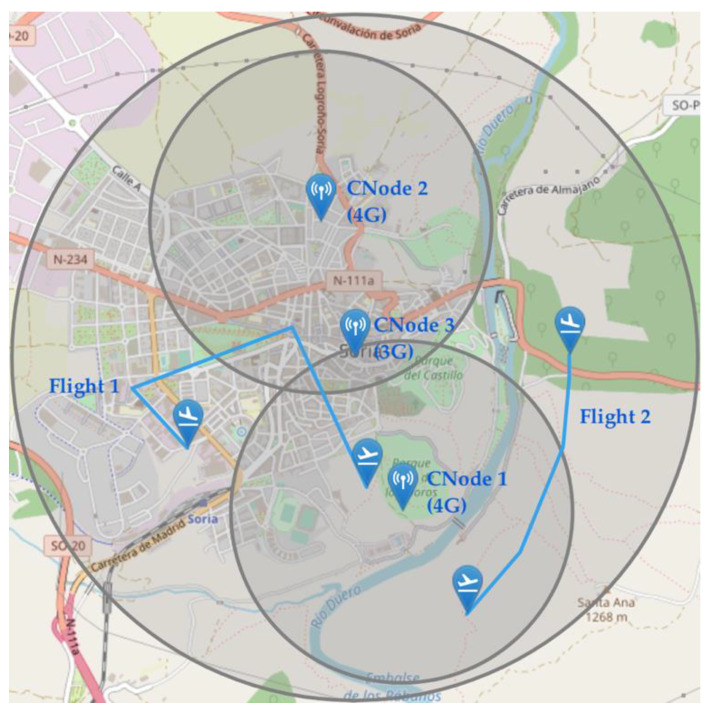
Scenario 6.3 with two different BVLOS flight plans and 3 different CNodes.

In this last scenario, we show the functioning of the communications network. The main evidence that the communications network is working properly is that it has already been able to carry traffic between the different actors in the previous scenarios. However, we propose the following scenario (Figure 18) to show that coverage and technology restrictions are respected and that BVLOS-type operations work as expected. This figure shows a simulation environment with three mobile communications nodes and their respective coverages: two 4G technology nodes (CNode1 and CNode2) and a 3G technology node (CNode3). In this environment, we simulate two BVLOS flights: Flight 1 which is carried out by a drone using exclusively 4G technology and flight 2 which is flown with a drone compatible with 3G technology.

**Figure 19:**
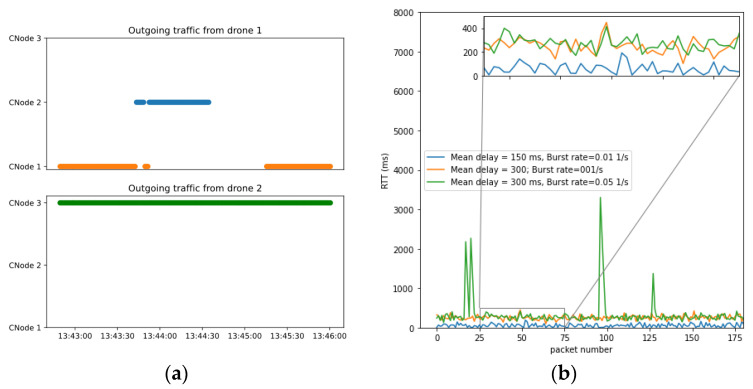
(**a**) Traffic processed by each CNode separated from its origin. (**b**) Delay experienced by UTM-received packages from flight 2 with three different network configurations.

**Figure 20:**
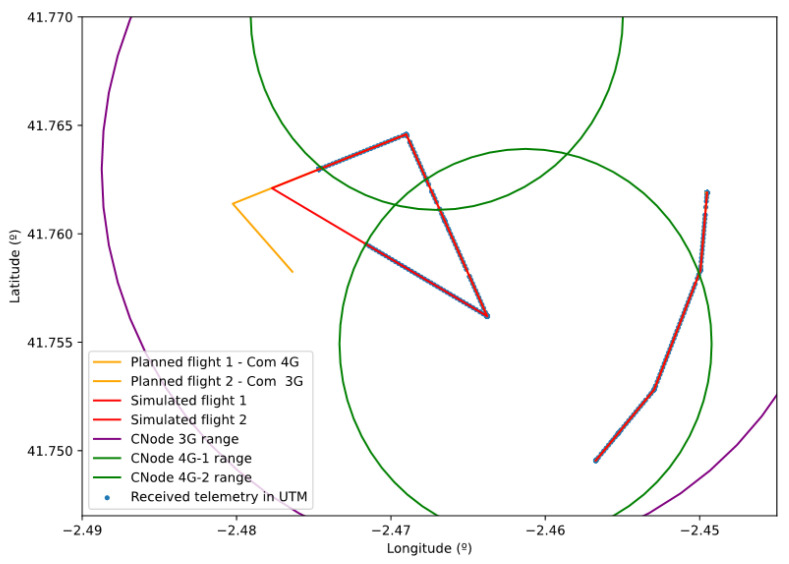
Simulated trajectory for each flight in this scenario along with the expected trajectory, received UTM telemetry and CNode ranges.

In Figure 19a, we can see which base stations process the C2 traffic of the two drones in the scenario. We can see that nodes 1 and 2 carry all the traffic from flight 1, since it is only compatible with 4G technology. The drone is assigned to either node depending on the distances to both drones, as long as it is within communication nodes coverage. In fact, we can see in Figure 20 that the drone is out of range of both 4G technology base stations in part of the operation. This implies that, since the drone is not compatible with 3G, node 3 cannot relay its traffic and the C2 link is interrupted. The drone detects this interruption and automatically initiates a return to home (RTH) maneuver. Since the communications are interrupted, the GCS does not receive telemetry messages from the drone so it cannot send position updates to the UTM system. However, while executing the RTH maneuver, the drone re-enters the coverage zone of a 4G node, automatically re-establishing communications, which results in the UTM resuming telemetry reception.

As for the second flight, we see that all the traffic is carried uninterruptedly by base station 3, which is the only one compatible with 3G technology, despite the existence of other closer base stations. At the same time, all simulated packets are experiencing the simulated communication disturbances. In Figure 19b, we show the delay experienced by UTM-received packages from flight 2 for three different network model configurations in three different executions. In all cases, we see a small variable delay for most of the packets (due to the simulated transmission delays) and some peaks for some consecutive packets, due to packet losses or bursts.

## 7. Conclusions and Future Work

The worldwide drone usage expansion demands the technological development of UTM systems. Due to their complexity and critical nature, it is necessary to evaluate UTM platforms in a wide variety of scenarios to verify its safe operation. Currently, these tests are performed by carrying out real flights that reproduce comprehensive scenarios. However, as we discussed in the introduction, this procedure is limited and not very scalable. The usual solution to solve these limitations is to resort to simulation techniques. As we depicted in Table 2, after analyzing existing literature, we can conclude that there are no complete simulation solutions available. More specifically, current solutions do not consider the impact of communication networks and surveillance sensors which are key for UTM systems functioning.

To solve this problem, we proposed a comprehensive agent-based simulation architecture that allows to simulate the behavior and interactions of the main UTM related agents: drones, pilots, ground control stations, surveillance sensors, and communication networks. The final goal of this platform is to provide to a set of simulated signals required by a UTM system in both pre-flight and in-flight stages, such as: flight plans, surveillance tracks, telemetry information, alerts, etc. This way, it is possible to perform a system-level evaluation of a UTM platform and analyze possible emergent behaviors.

This architecture is based on microservices and provides a series of abstractions and mechanisms that allow modeling the behavior of such agents in a simple, reusable, and extensible way. It also allows exchanging events and messages between agents in order to simulate their interactions. Moreover, the proposed simulator generates a common simulation environment for the simulated agents and the UTM platform including weather, terrain, and airspace information. It also allows manually scheduling contingencies (in addition to those arising naturally during agent’s interactions) so that the UTM system can be tested in the presence of unexpected behaviors. Based on this architecture, we have implemented a simulator where we have modeled each one of the actors with a sufficient level of detail to be able to generate the aforementioned input signals.

During the validation of the system, we demonstrated that it is possible to use this architecture to simulate complex scenarios in which several agents interact in concurrence of unforeseen situations that are forced by the user. We also verified the correct functioning of the UTM-simulator interconnection mechanisms and those envisioned to provide information to user-defined evaluation services. To conclude, the proposed simulator constitutes a valuable complement and, ultimately, an alternative to the use of real tests for the validation of UTM systems. Its implementation results in a reduction of the development costs of these systems and an improvement in the achieved levels of operational safety.

In order to improve the use of the simulator, additional development efforts are planned, some of which have already been introduced. Currently, UTM feedback information is not used during the flight phase as we have designed an open loop system. Clearly, the behavior of the pilots, who ultimately determine the behavior of the drones, is not unrelated to the information received from the UTM system, such as alerts. Therefore, it would be appropriate to extend the presented simulator to take into account the output information produced by the UTM system, thus converting the simulator-UTM platform system into a closed-loop system. Another point that can limit the final usefulness of the simulator is the design decision to use real time simulation. In order to allow accelerated time simulation, it would be necessary to create a common timing layer to the simulation environment and to the UTM systems that would allow accelerating or eliminating interim between events and that could be integrated in UTM systems in a simple way. Additionally, the proposed simulator has not considered the presence of manned aircraft or its interconnection with traditional ATM systems. Therefore, it would be interesting to integrate the proposed simulator with other existing simulators for ATM systems in order to verify UTM-ATM use cases. Finally, potential improvements in specific agents’ simulation models could result in an even richer and more realistic simulation environment, taking advantage of the already mature agent-based simulation platform presented.

## Figures and Tables

**Table 1:** European U-Space functionalities, expected deployment year, and main functionality deployed in each phase.

**U1—2019** VLOS (visual line of sight) operations	Electronic drone registration.
	Remote UAVs identification.
	Provision of static geofences (no drone zones) to users.
**U2—2022** BVLOS (beyond visual line of sight) operations	Flight plan authorization (including strategical deconfliction of flight plans).
	Drone tracking from multiple information sources.
	Air safety monitoring (no incursion in geofences, terrain avoidance, conflicts between aircraft, etc.).
	Flight plan conformance monitoring (compliance of flight with authorized flightplan).
	Weather provision to operators.
	Access to aeronautic and Air Traffic Control (ATC) data.
	Emergency management.
	Provision to pilots of nearby traffic
**U3—2027** Urban mobility and high flight density	Dynamic (on-board) geofencing.
	Improved interface with Air Traffic Management (ATC/ATM).
	Tactical (in-flight) conflict detection and resolution.
	Drone congestion management.
**U4—2035** Full ATM-UTM integration	Additional services.

**Table 2:** Elements considered (✔) in related work and in our proposed simulator.

	Simulated Behaviors	Proposed Simulator	Hentati et al. [21]	Al-Mousa et al. [23]	Rodr.-Feran. et al. [24]	Zhao et al. [25]	Homola et al. [26]
Drone	Cooperative/non-cooperative sensors	✔					
	Drone trajectory	✔	✔	✔	✔	✔	✔
	Control error	✔					
	Navigation error	✔					
	Pilot actions	✔	✔	✔			✔
	Contingencies response	✔					
GCS	VLOS C2 link	✔	✔	✔			
	BVLOS C2 link	✔		✔		✔	
	Orders and telemetry sending over C2 link	✔					
	UTM system interaction	✔					✔
	Contingencies response	✔					
Sensors	Detection by non-cooperative sensors	✔					
	Detection by cooperative sensors	✔					
	Detection errors (precision, false alarms, etc.)	✔					
	Plot processing to generate tracks	✔					
	UTM system interaction	✔					
	Contingencies response	✔					
Network	Routing and forwarding	✔		✔			
	Packets disturbances	✔		✔			
	Mobile communication nodes with range	✔				✔	
	Compatibility with communications technologies	✔					
	Contingencies response	✔					
Environment	Airspace definition	✔			✔		
	Weather information	✔	✔	✔			
	Realistic terrain model	✔	✔				✔

## Data Availability

Data sharing not applicable.
